# Mixture modeling of microarray gene expression data

**DOI:** 10.1186/1753-6561-1-s1-s50

**Published:** 2007-12-18

**Authors:** Yang Yang, Adam P Tashman, Jung Yeon Lee, Seungtai Yoon, Wenyang Mao, Kwangmi Ahn, Wonkuk Kim, Nancy R Mendell, Derek Gordon, Stephen J Finch

**Affiliations:** 1 Department of Applied Mathematics and Statistics, Stony Brook University, Stony Brook, New York 11790, USA; 2 Cold Spring Harbor Laboratory, Cold Spring Harbor, New York 11724, USA; 3 Department of Health Evaluation Sciences, A210, Penn State College of Medicine, 600 Centerview Drive, Hershey, Pennsylvania 17033, USA; 4 Department of Genetics, Rutgers University, 145 Bevier Road, Room 128, Piscataway, New Jersey 08854, USA

## Abstract

About 28% of genes appear to have an expression pattern that follows a mixture distribution. We use first- and second-order partial correlation coefficients to identify trios and quartets of non-sex-linked genes that are highly associated and that are also mixtures. We identified 18 trio and 35 quartet mixtures and evaluated their mixture distribution concordance. Concordance was defined as the proportion of observations that simultaneously fall in the component with the higher mean or simultaneously in the component with the lower mean based on their Bayesian posterior probabilities. These trios and quartets have a concordance rate greater than 80%. There are 33 genes involved in these trios and quartets. A factor analysis with varimax rotation identifies three gene groups based on their factor loadings. One group of 18 genes has a concordance rate of 56.7%, another group of 8 genes has a concordance rate of 60.8%, and a third group of 7 genes has a concordance rate of 69.6%. Each of these rates is highly significant, suggesting that there may be strong biological underpinnings for the mixture mechanisms of these genes. Bayesian factor screening confirms this hypothesis by identifying six single-nucleotide polymorphisms that are significantly associated with the expression phenotypes of the five most concordant genes in the first group.

## Background

McLachlan et al. [1] introduced a mixture analysis approach to the clustering of microarray expression data, in particular, of tissue samples on a very large number of genes. Maclean et al. [2] developed the SKUMIX algorithm, which can test whether a mixture model fits the genetic data with skewness removed by Box-Cox transformation [3], and then used a likelihood-ratio test (LRT) statistic to determine whether the two-component model appears to fit the data better than the single-component model. Given the high degree of correlation among the gene expression variables, Simon's work [4] suggests that one use first- and second-order partial correlation coefficients to find trios and quartets of genes that have high degrees of "explanation." Here we focus on trios and quartets comprising only non-sex-linked genes that appear to follow a mixture distribution to explore the associations of these mixing mechanisms. For example, if there is one common mixture mechanism governing all of the genes in a set, then the fraction of subjects simultaneously falling in the same mixing component of these genes would be high. We then use varimax factor anlaysis [5,6] to see whether we can identify more than four genes operating under a common mixing mechanism. One confirmation that the common mixture mechanism has biological importance would be to identify genetic relationships between a subject's single-nucleotide polymorphism (SNP) genotypes and expression phenotypes. Bayesian factor screening (BFS) [7] is one statistical strategy proposed to identify these relations. In this paper, the mixture model-based approach with extended SKUMIX algorithm, partial correlation analysis, factor analysis, and BFS are systematically combined to analyze the Problem 1 data set in Genetic Analysis Workshop 15 (GAW15) [8,9].

## Methods

### Box-Cox family of transformations

Given the expression intensities *x*_1, *j*_, *x*_2, *j*_,..., *x*_*n*, *j *_(n = 194) for the *j*^th^(*j *= 1, 2,..., 3554) gene, the Box-Cox family [3] transforming *x*_*j *_= (*x*_1, *j*_, *x*_2, *j*_,..., *x*_*n*, *j*_) to $x_{j}^{\left(p_{j}\right)}=(x_{1,j}^{\left(p_{j}\right)},x_{2,j}^{\left(p_{j}\right)},...,x_{n,j}^{\left(p_{j}\right)})$ with power parameter *p*_*j *_is:

$$x_{j}^{\left(p_{j}\right)}=\{\begin{array}{ll} (x_{j}^{p_{j}}-1)/p_{j}, & p_{j}\neq 0\\ ln(x_{j}), & p_{j}=0 \end{array}.$$

The expression intensities transformed here are the original observations rather than the log_2 _values reported in the data set. The 0.3-power transformation is the transformation that maximizes the probability plot correlation coefficient (PPC, see Filliben [10]) for the greatest number of genes.

### Mixture analysis using Gaussian mixture model

The SKUMIX algorithm is extended in our mixture analysis. First, we applied the Box-Cox family of power transformations without the scale parameter (see Eq. (1)). Second, we considered a wider interval [0, 1.5] than the one recommended by Maclean et al. [2] for selecting the optimal power parameter. Third, as suggested by Ning et al. [11], we used 6.9 as the 0.05 critical value for LRT of "a single component distribution" vs. "a mixture distribution of two components."

### Partial correlation analysis

We calculate the Pearson product moment correlation coefficients *r*_*ij *_= *r*(*x*_*i*_, *x*_*j*_), first-order partial correlations *r*_*ij*.*k *_= *r*(*x*_*i*_, *x*_*j*_|*x*_*k*_) and second-order partial correlation coefficients *r*_*ij*.*kl *_= *r*(*x*_*i*_, *x*_*j*_|*x*_*k*_, *x*_*l*_) [12] for expression phenotype variables whose values are the 0.3-power Box-Cox transformed expressions. The partial correlation criteria are:

$$T:|r(x_{i},x_{j})|>0.8\;and\;r_{ij}^{2}-r_{ij.k}^{2}>0.63\;for\;i\neq j\neq k$$

$$Q:|r_{ij}|>0.8,\;r_{ij}^{2}-r_{ij.kl}^{2}>0.63,\;r_{ij}^{2}-r_{ij.k}^{2}<0.63,\;and\;r_{ij}^{2}-r_{ij.l}^{2}<0.63\;for\;i\neq j\neq k\neq l.$$

The last two inequalities in criterion Q reduce redundancy by removing quartets built on trios. We identify trios of expression phenotype variables (*x*_*i*_, *x*_*j*_, *x*_*k*_) that meet criterion T and quartets (*x*_*i*_, *x*_*j*_, *x*_*k*_, *x*_*l*_) that meet criterion Q.

### Measure of common mixing mechanism

When a gene expression variable appeared to be a mixture, we fit a mixture of two Gaussian components with equal variance using MCLUST [13] and classified each subject into the component with the largest Bayesian posterior probability [14]. We called the component with estimated probability less than 0.5 the "uncommon component" and the other one the "common component." The *concordance rate *(*C*) in a gene set is the ratio of subjects that simultaneously fall into the uncommon or the common components for all the genes in the set. A value of *C *close to 1 suggests a common mixture mechanism. We selected genes in a trio or quartet with *C *≥ 80% for the factor analysis. Fleiss' statistic *κ *[15] was used to assess agreement. A value of *κ *> 0.75 indicated excellent agreement, while *κ *< 0.40 indicated poor agreement [16].

### Factor analysis

Each gene expression variable that appeared to be a mixture and was present in one or more trios or quartets was included in a factor analysis using varimax rotation.

### Bayesian factor screening

We used BFS [7,17] to identify SNPs significantly associated with expressions of the genes from the factor analysis. We only considered the regression model with second-order interactions:

$$y=\alpha +\sum_{j=1}^{S}\beta_{j}x_{j}+\sum_{i<j}\beta_{ij}x_{i}x_{j}+\varepsilon ,$$

where the values of *x*_1_, *x*_2_,..., *x*_*S *_are recoded genotypes (1 for minor homozygotes, 2 for heterozygotes, 3 for major homozygotes, and -2 for missing data) of *S *(2682) consistent and informative SNPs that may have linear main effects and/or interaction effects on the gene expression variable *γ*. Let *γ *be the indicator vector such that *γ*_*j *_= 0 if *β*_*j *_= 0 and *β*_*ij *_= 0 for all *i *≠ *j*, and *γ*_*j *_= 1 if otherwise. Then a model (or an element) in the model space can be represented by a binary vector *γ *= (*γ*_1_, *γ*_2_,..., *γ*_*S*_) that ranges from *γ*^(1) ^= (0, 0,..., 0) to $\gamma^{\left(2^{\text{S}}\right)}$ = (1, 1,..., 1), with the model size defined as $m=\sum_{j=1}^{S}\gamma_{j}\in [0,S]$. In our study, we set the model size *m *= 6, the chain length *CL *= 200,000, and the magnitude of the effect relative to the experimental noise *λ *= 1.5. We use the Java program developed by Yoon [17] to find the optimal model from the model subspace consisting of $C_{6}^{2,682}$ = 5.14 × 10^17 ^elements. The output gives an estimate of each SNP's marginal posterior probability (MPP) of appearing in the 200,000 selected models. An MPP close to 1 suggests that the SNP is an important factor (either as a main effect or as one of two terms in an interaction) for the gene expression variable.

## Results

**Figure 1:**
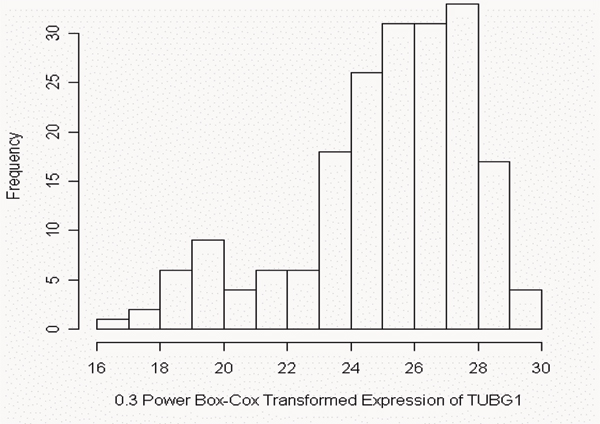
Histogram of the 0.3-power Box-Cox transformed *TUBG1*.

Of the 3554 gene expressions analyzed, 2561 appear to follow a normal distribution. After a Box-Cox transformation to maximize the PPC, 659 give evidence of being a mixture with two components, and 334 appear to have three components. Figure 1 contains the histogram of the 0.3-power Box-Cox transformed expressions of TUBG1 that appears to follow a mixture of two components. The left component is the uncommon one, with estimated proportion 15.7% and estimated mean of 19.9. The right component is the common one, with estimated proportion 84.3% and estimated mean of 26.1.

We find 233 trios containing 54 genes that meet criterion T, and 115,840 quartets containing 3554 genes that meet criterion Q. Of the 233 trios, 88 include only mixture distributions (involving a total of 29 genes). Of the 115,840 quartets, 7342 include only mixture distributions (involving a total of 902 genes). A number of trios and quartets contain only sex-linked genes. When we exclude these sex-linked gene sets, there are 18 trios and 35 quartets with a value of *C *≥ 80%. These trios and quartets contain 33 non-sex-linked genes in total.

One example is the quartet containing HNRPAB, PSMD2, TUBG1, and AHSA1. Each of these genes appears to be a mixture with very small *p*-value; Figure 1 is the histogram of the 0.3-power transformed expressions of TUBG1. The correlation between PSMD2 and HNRPAB (using the 0.3-power transformed expressions) is 0.825, and the partial correlation between PSMD2 and HNRPAB controlling for TUBG1 and AHSA1 is 0.094. Table 1 is the four-way contingency table in which each subject is classified by the Bayesian posterior probability into the common or uncommon component. The *C *value for this set of genes is 83.50%. Specifically, 136 of 194 subjects are simultaneously common and 26 are simultaneously uncommon in these four genes so that 162 of 194 subjects (that is, 83.50%) are concordant. There are, respectively, 14 (1+6+7) and 9 (6+3) additional subjects that fall into the common and uncommon components of three genes out of the four, suggesting a larger concordance rate for smaller gene sets.

**Table 1:** Contingency table for *TUBG1*, *AHSA1*, *PSMD2*, and *HNRPAB*

Subjects Classified into Common or Uncommon Components						
*TUBG1*↓	*AHSA1*↓	*PSMD2 *→	Common		Uncommon	
		
		*HNRPAB *→	Common	Uncommon	Common	Uncommon

Common	Common		136	6	7	4
	Uncommon		1	3	2	6
						
Uncommon	Common		0	0	0	3
	Uncommon		0	0	0	26

A factor analysis on the 0.3-power transformed gene expression levels of the 33 non-sex-linked genes identifies three factor groups. As listed in Table 2, Factor 1 appears to consist of 18 genes, Factor 2 appears to consist of 8 genes, and Factor 3 appears to consist of 7 genes. A trio with a high value of *C *contains genes from Factor 2 or from Factor 3. A quartet with a high value of *C *contained all genes either from Factor 1 or from Factor 3.

**Table 2:** Concordance rate (*C*) for sets of genes selected from factors

	Factor 1			Factor 2			Factor 3		
			
Seq	Gene	*C *(%)	*κ*	Gene	*C *(%)	*κ*	Gene	*C *(%)	*κ*
1	AHSA1, ELAC2	94.85	0.8257	RPL32, RPS18	92.78	0.8278	PRKAR1A, ST13	93.30	0.8609
2	CCT3	90.72	0.7773	RPS15	87.63	0.8104	MATR3	86.08	0.8117
3	TUBG1	88.66	0.7663	RPS28	81.96	0.7701	PPM1B	80.93	0.7934
4	TACC3	85.57	0.7555	RPS10^a^	76.29	0.7081	PDCD10	77.84	0.7825
5	NDUFS6	82.99	0.7495	RPS19	72.68	0.6677	SF3B1	73.71	0.7684
6	CDC45L	80.41	0.7273	B2M	68.56	0.6340	G3BP2	69.59	0.7521
7	DHX9	77.84	0.7216	RPS10^b^	64.43	0.5578	NA	NA	NA
8	FEN1	75.26	0.7096	PABPC1	60.82	0.5510	NA	NA	NA
9	HNRPAB	73.71	0.7070	NA	NA	NA	NA	NA	NA
10	PSMD2	72.16	0.7034	NA	NA	NA	NA	NA	NA
11	CSE1L	70.10	0.6931	NA	NA	NA	NA	NA	NA
12	C20orf24	67.53	0.6800	NA	NA	NA	NA	NA	NA
13	JTV1	65.46	0.6755	NA	NA	NA	NA	NA	NA
14	LANCL2	63.92	0.6662	NA	NA	NA	NA	NA	NA
15	TSTA3	62.37	0.6572	NA	NA	NA	NA	NA	NA
16	CCT7	59.79	0.6514	NA	NA	NA	NA	NA	NA
17	SOD1	56.70	0.6440	NA	NA	NA	NA	NA	NA

^a^Measured on probe set 200095_x_at

^b^Measured on probe set 200817_x_at

We then examined whether the genes in each factor group follow a common mixture mechanism. In each factor group, we started with the pair of genes that have the highest *C *value and added the gene from the factor group that least reduces *C*. For example, the first two genes *A*_1 _= {AHSA1, ELAC2} have the largest *C *= 94.85%, with *κ *= 0.8257. The gene *CCT3 *had the least reduction in *C *value of the 16 genes remaining in Factor 1. We include genes from Factor 1 sequentially until we got the final gene group *A*_17 _= *A*_16 _∪ {SOD1} with *C *= 56.70% and *κ *= 0.6440. For this factor group, the reduction in *C *value with adding one gene to the set ranges from 1% to 3%. Similar results hold for Factor groups 2 and 3.

We extended the mixture analysis with BFS applied to the five most concordant genes in Group 1 (*AHSA*1, *ELAC*2, *CCT*3, *TUBG1*, and *TACC*3, with *C *> 85% and *κ *> 0.75). For each of these genes, BFS identifies six SNPs that have very large MPPs, as shown in Table 3.

**Table 3:** Marginal posterior probabilities (MPPs) of six SNPs associated with *AHSA1*, *ELAC2*, *CCT3*, *TUBG1*, and *TACC3*

SNP/Location	*AHSA1*	*ELAC2*	*CCT3*^a^	*TUBG1*	*TACC3*
rs1438676/chr 5	0.9976	0.9976	0.3024	0.5618	0.9976
rs1560143/chr 5	0.9976	0.9976	0.3024	0.5618	0.9976
rs1453389/chr 11	0.9976	0.9976	0.3024	0.5618	0.9976
rs1945465/chr 11	0.9976	0.9976	0.3024	0.5618	0.9976
rs1993205/chr 11	0.9976	0.9976	0.3024	0.5618	0.9976
rs2043041/chr 18	0.9992	0.9992	0.9992	0.5633	0.9992

^a^There are five SNPs not reported here with MPP between 0.3024 and 0.9992. These five SNPs do not have MPPs higher than the ones reported for genes *AHSA1*, *ELAC2*, *TUBG1*, and *TACC3*.

## Conclusion

About 28% of genes from GAW15 Problem 1 appear to follow a two- or threecomponent mixture distribution. Important structural relations seem to be partially disentangled using first- and second-order partial correlation matrices. These partial correlation coefficients can be effectively used to identify trios and quartets of genes that have a more complex structure. There are 18 trios and 35 quartets in which the genes are all non-sex-linked but follow a common mixture distribution with *C *≥ 80%. That is, the underlying mixture mechanisms of these genes appear to be highly associated. This pattern of association appears to involve a large number of genes. A computational strategy using the varimax rotation in a factor analysis finds a group of 18 genes with *C *= 56.7%, another group of 9 genes with *C *= 60.8%, and a third group of 7 genes with *C *= 69.6%. The R package MIXMECH that has been developed here for the mixture analysis of microarray expression data is freely available at the websites  and .

The significance of these findings is not immediately clear. For example, one possible source of a mixture mechanism that is not substantively interesting is the non-homogenous measurement process of the gene expressions. The data used here are from 14 pedigrees rather than from a random sample of cases or controls. Therefore, we do not know the magnitude of the effect of dependence among subjects generated by the family structure. In results not shown here, however, we obtained results parallel to these when we restricted our analysis to the 56 unrelated founders, suggesting that the effect of the intra-familial dependence is minor. As always, replication of these results on an independent data set is a crucial step to confirm the scientific value of this approach and our findings.

Nevertheless, the high concordance rates and high Fleiss *κ *coefficients suggest that there may be a common mechanism determining which component a subject falls into. More importantly, the BFS result showing a strong association between the five most concordant genes in Group 1 with the six SNPs strongly suggests that there is an underlying biological mechanism.

## Competing interests

The author(s) declare that they have no competing interests.

## References

[B1] McLachlan GJ, Bean RW, Peel D (2002). A mixture model-based approach to the clustering of microarray expression data. Bioinformatics.

[B2] Maclean CJ, Morton NE, Elston RC (1976). Skewness in commingled distributions. Biometrics.

[B3] Box GEP, Cox DR (1964). An analysis of transformations. J R Stat Soc Ser B.

[B4] Simon HA (1954). Spurious correlation: a causal interpretation. J Am Stat Assoc.

[B5] Gorsuch RL (1983). Factor Analysis.

[B6] McLachlan GF, Peel D, Bean RW (2003). Modelling high dimensional data by mixtures of factor analyzers. Comput Stat Data Anal.

[B7] Yoon S, Suh YJ, Mendell NR, Ye KQ (2005). A Bayesian approach for applying Haseman-Elston methods. BMC Genetics.

[B8] Morley M, Molony CM, Weber TM, Devlin JL, Ewens KG, Spielman RS, Cheung VG (2004). Genetic analysis of genome-wide variation in human gene expression. Nature.

[B9] Cheung VG, Spielman RS, Ewens KG, Weber TM, Morley M, Burdick JT (2005). Mapping determinants of human gene expression by regional and genome-wide association. Nature.

[B10] Filliben JJ (1975). The probability plot correlation coefficient test for normality. Technometrics.

[B11] Ning YM, Finch SJ (2000). The null distribution of the likelihood ratio test for a mixture of two normals after a restricted Box-Cox transformation. Comm Stat Simul Comp.

[B12] Sokal RR, Rohlf FJ (1995). Biometry.

[B13] Fraley C, Raftery AE (2002). Model-based clustering, discriminant analysis, and density estimation. J Am Stat Assoc.

[B14] Casella G, Berger RL (1990). Statistical Inference.

[B15] Fleiss JL (1971). Measuring nominal scale agreement among many raters. Psychol Bull.

[B16] Fleiss JL (1981). Statistical Methods for Rates and Proportions.

[B17] Yoon S (2006). Bayesian factor screening. Dissertation.

